# Evaluating the Linkage Between Resistin and Viral Seropositivity in Psoriasis: Evidence from a Tertiary Centre

**DOI:** 10.3390/life15071054

**Published:** 2025-06-30

**Authors:** Habeeb Ali Baig, Waseema Sultana, Mohamed Soliman, Dhaifallah Alenizi, Awwad Alenezy, Srinath Mote, Ahmed M. S. Hegazy, Bader Khalid Alanazi, Mansour Srhan Alanazi, Yousef Albedaiwi, Nawal Salama Gouda

**Affiliations:** 1 Department of Microbiology, Faculty of Medicine, Northern Border University, Arar 91431, Saudi Arabia; docbaig@yahoo.com (H.A.B.); mohamed.sherif@nbu.edu.sa (M.S.); nawalsalama@gmail.com (N.S.G.); 2 ICMR/DHR—Viral Research and Diagnostic Laboratory, Department of Microbiology, Osmania Medical College, Hyderabad 500079, Telangana, India; waseemasultana6@gmail.com; 3 Department of Medicine, Faculty of Medicine Northern, Border University, Arar 91431, Saudi Arabiadrbaderalanazi@gmail.com (B.K.A.); mansour.srhan.g@gmail.com (M.S.A.); 4 Department of Family and Community Medicine, Faculty of Medicine, Northern Border University, Arar 91431, Saudi Arabia; dr.awwad@hotmail.com; 5 Department of Anatomy, Faculty of Medicine, Northern Border University, Arar 91431, Saudi Arabia; ahmed0562301954@yahoo.com; 6 Department of Dermatology, Faculty of Medicine, Northern Border University, Arar 91431, Saudi Arabia; youbed@gmail.com

**Keywords:** psoriasis, viral infections, ELISA, immune-mediated inflammation, resistin, viral seropositivity

## Abstract

Psoriasis, a chronic immune-mediated inflammatory skin disorder, presents complex pathogenetic mechanisms potentially influenced by viral infections. This comprehensive study explored the possible interplay of resistance and viral infections among psoriasis patients using serological screening techniques. The investigation involved 90 patients aged 23–45 years, systematically examining viral seropositivity for HSV (herpes simplex virus), HZ (herpes zoster), HBV (hepatitis B virus), HIV (human immunodeficiency virus), and HCV (hepatitis C virus) through ELISA testing. The findings revealed notable active or recent viral infection rates: 8.9% HSV positivity, 2.2% HZ antibody detection, 4.4% HCV positivity, and 4.4% HIV positivity. The research can contribute to current knowledge gaps, broaden the knowledge regarding the relationship between psoriasis and viral infection, and assess resistance, as it can mediate the interaction. The results can lead to improved diagnosis, treatment, and patient care options. This study emphasizes the importance of thorough viral testing for psoriasis patients, as well as focused therapeutic regimens that take into account viral co-infections. It elucidates the complex networks of biological relationships between immune factors, contributes information that is critical to our understanding of the multifactorial etiology of psoriasis, and concludes with a strong argument for investigating the mechanisms of viral involvement in this chronic-relapsing inflammatory disease.

## 1. Introduction

Psoriasis is a chronic inflammatory disease, associated with immune system dysregulation, though the precise cause remains unknown. It is associated with abnormal keratinocyte growth and differentiation, and with skin inflammation characterized by the infiltration of immune cells (lymphocytes, T-cells, macrophages, neutrophils) and a host of other inflammatory mediators [1]. The underlying process in psoriasis is a perpetual loop in which overactive keratinocytes interact with immune cells that infiltrate the skin. Furthermore, the activation of autoreactive T lymphocytes by autoantigens is hypothesized to play an important role in disease initiation and progression [2,3]. Plaque psoriasis (psoriasis vulgaris) is the most common form of the disease, and is characterized by the appearance of raised, dry, itchy patches of skin covered with silver-colored scales [4]. Plaques can develop anywhere on the body, but are most frequently found on the scalp, elbows, knees and lower back [5,6]. The symptoms associated with plaque psoriasis may result in severe discomfort, including itching and pain, and may negatively impact quality of life [7]. It is one of the most common autoimmune diseases around the world, affecting an estimated 2–4% of the population [8]. The condition has a higher prevalence in certain regions or populations of countries with cooler climates than tropical regions [9]. Psoriasis typically presents in ages 15–35, affecting men and women equally. The disease can be severe in a few ethnic groups potentially due to genetic and environmental factors [10]. Psoriasis affects a patient’s emotional well-being alongside its physical impacts. Ethnic group variance, predominantly observed more in Western countries, makes it distinct because it has a genetic basis and is inherited through multiple genes [11].

Resistin, a key member of the resistin-like molecule (RELM) family, is a small protein with a cysteine-rich C-terminal domain that is highly conserved. It is primarily released by various leukocytes, including monocytes, macrophages, and neutrophils, in response to pro-inflammatory cytokines such as TNF-α and interleukin (IL)-6 [12,13,14]. This 11 kDa protein, which contains 12 cysteine residues, functions as a polypeptide hormone. Recent studies have also detected resistin secretion by keratinocytes and sebaceous glands within the skin [15]. The gene encoding resistin is located on chromosome 19p13.3, and in humans, the protein consists of 108 amino acids. In circulation, resistin is found primarily as an oligomer (>660 kDa) and a trimer (45 kDa) [16,17]. The oligomeric form of resistin exhibits potent pro-inflammatory activity [18,19]. The protein undergoes a concentration-dependent conformational change from an α-helix to a β-sheet structure, facilitated by intermolecular and intramolecular disulfide bonds, which leads to the formation of its oligomeric state at higher concentrations [20]. Serum levels of resistin typically range from 7 to 22 ng/mL [13].

Resistin has been shown to promote the release of inflammatory cytokines, including IL-2, IL-6, and TNF-α [21,22]. In the presence of inflammatory stimuli like lipopolysaccharide (LPS), TNF-α, IL-1β, and IL-6, monocytes and macrophages increase resistin gene expression [23,24]. These inflammatory mechanisms play a key role in autoimmune disorders, viral infections, and cancers [25]. In psoriasis, resistin is involved in the disease’s progression and development via the release of pro-inflammatory cytokines including IL-6, IL-2, and TNF-α [21,22]. Increased levels of resistin in psoriatic patients are associated with disease severity [26,27]. In addition, patients with psoriatic disease have higher levels of resistin, C-reactive protein (CRP), and cytokine macrophage inflammatory protein (CMIT), which leads to an increased risk of cardiovascular disease and metabolic syndrome [28]. Resistin also promotes TNF-α production and the secretion of CXCL8 by monocytes, promoting keratinocyte proliferation and T lymphocyte recruitment to the dermis. Consequently, resistin has a strong link with TNF-α, a major inflammatory mediator in psoriasis [29,30]. Furthermore, resistin may contribute to the dysfunction of Foxp3+ Treg cells by impairing their tolerance to self-antigens, which exacerbates the autoimmune response and facilitates psoriasis development [31,32]. The presence of viral infection in psoriasis patients may affect the severity as well as progression of psoriasis, as both cause considerable immune activation [33]. Improved patient outcomes may result from effective treatment strategies based on the prevalence of viral infections in this subgroup. Concurrent viral infection may make these risks worse. To avert serious consequences and implement comprehensive risk management in patients, it is essential to diagnose viral infections. Monitoring psoriasis patients with elevated inflammatory markers and the prevalence of viral infections can help guide screening recommendations and public health initiatives [34].

Psoriasis is a complex condition that goes beyond the skin, often affecting multiple organ systems. The condition is known to have a huge impact on the immune system, leading to an exaggerated inflammatory state; however, the relationship with other autoimmune and chronic diseases is being explored. The onset and exacerbation of psoriasis can be influenced by various environmental and lifestyle factors [35]. For example, dietary habits, exposure to pollution, and physical trauma are known to act as potential triggers for flare-ups. These factors can interact with the immune system and accelerate the inflammatory process, leading to the worsening of psoriasis symptoms. The recent advances in imaging and diagnostic procedures have advanced the ability to assess disease progress and treatment responses better, providing insight into the underpinning mechanisms [36]. The disease has been shown to have an increased risk of comorbidities conditions such as cardiovascular disease, which emphasizes the need for comprehensive management approaches. As therapies and treatments evolve, medical tourism and personalized therapy are increasingly focusing on the development of targeted biologics and small molecule inhibitors to enhance treatment effectiveness [37]. Along with the therapies, the support people receive from being around others, social support, and an emphasis on education have continued to provide an improved quality of life with psoriasis [38].

HCV (Hepatitis C virus) infection is associated with several extrahepatic manifestations, which affect multiple organ systems beyond the liver. These include autoimmune disorders such as porphyria cutanea tarda, lichen planus, arthritis, sicca syndrome, nephropathies, thyroid disease, lung fibrosis, and metabolic abnormalities [39]. Various mechanisms have been proposed to explain the connection between HCV infection and psoriasis [40,41]. Additionally, studies indicate that individuals with Human Immunodeficiency Virus (HIV) experience a higher prevalence of psoriasis compared to the general population [42]. Antiretroviral medications have been demonstrated to alleviate psoriatic symptoms in these patients [43]. The Herpes Simplex Virus (HSV) is known to elicit psoriasis flares; nevertheless, research indicates that controlling HSV infections in psoriasis patients could help attenuate psoriatic symptoms [44]. The relationship between HSV and psoriasis reinforces the need to monitor and treat viral infection in these patients [45]. The literature reports higher incidence of herpes zoster (HZ) among psoriasis patients due to existing immunological dysregulation and immunosuppressive medicines. At the same time, individuals experiencing HZ can have psoriatic flares as a result of the overproduction of cytokines and activation of T-cells [46]. The greatest impact of herpes zoster (HZ) is ongoing, severe cutaneous involvement; widespread, multidermatomal distribution, and in some, systemic dissemination of varicella-zoster virus (VZV). These findings are mostly likely applicable to the immunocompromised population in general, which includes people living with HIV, solid organ and bone marrow transplant donors/recipients, and immunosuppressive drugs [47]. Patients with psoriasis often receive a multi-drug regimen of biologics and methotrexate. Therefore, methotrexate has hepatotoxic and myelosuppressive toxicity, and biologics increase the risk of contracting the varicella-zoster virus at the same time [48]. Given the potential link between psoriasis and viral infection, and the significance of inflammatory markers like resistin, CRP, and CMIT in suggesting systemic inflammation, it is critical to explore the incidence of viral infection in psoriasis patients with elevated levels of these markers. This research could offer crucial insights into the underlying mechanisms of psoriasis, improve diagnostic precision, and lead to better therapeutic outcomes by enabling more personalized treatment strategies. The research can address existing knowledge gaps and enhance the understanding of the relationship between psoriasis and viral infections, while also evaluating the role of resistin in this interaction. The findings may contribute to improved diagnostic methods and more effective treatments in ultimately better patient care options.

### Research Aim

The research investigates the role of resistin in the immune response of patients with psoriasis, especially its modulation of the level and progression of viral co-infection with HSV, HIV, and HCV. Serums with active viral co-infection can be analyzed for resistin, as well as clinical documentation of viral load, as resistin levels can be increased as the viral load increases, resulting in a worsening of physical symptomology (i.e., psoriasis flare). Its resistin implications can also be researched through its interaction with immune cells and inflammatory mediators that promote psoriasis. The goal of this immunology research is to evaluate the link between viral infections and psoriasis flares, aiming to improve patient management and provide more effective treatment strategies from an immunological perspective.

## 2. Materials and Methods

**Figure 1:**
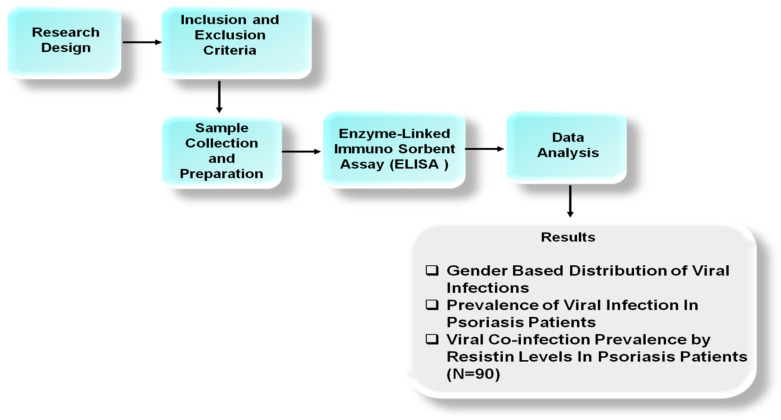
The working flow of methodology.

The research was conducted on patients with defined viral infections who had provided blood samples for analysis. It used samples to measure resistin levels through ELISA analyses and examined viral seropositivity through diagnostic tests for herpes simplex, hepatitis C, Human Immunodeficiency Virus, herpes zoster, and hepatitis B viruses. The resistin levels were classified into three categories (i.e., elevated, normal, and low) to understand how they were varied for viral illness. Statistical analyses were performed to examine the correlation between viral seropositivity and resistin levels, focusing on determining whether specific viral infections were linked to elevated resistin levels, indicating a systemic inflammatory response. Figure 1 presents the working flow of the methodology.

### 2.1. Research Design

The research utilized a cross-sectional design and examined 90 psoriasis patients (ages 23–45 years), thus providing a representative sample of patients with chronic inflammatory skin disease. The primary aim of the research was to describe the relationship between viral infections and resistin levels in patients with chronic inflammatory skin disease and to assess how these variables can interact with one another and contribute to disease progression. The research performed serological screening for multiple viral infections including HSV, HIV, HCV, and HZ to assess viral seropositivity in patients. The research measured resistin levels, an adipokine associated with inflammation, to examine whether there is an association between viral infections and pathological resistin, thus providing potentially mixed evidence of an association regarding viral infections. The research design was inclusive of other factors influencing the patients’ disease course such as medical history and treatment regimens, assisting in the development of possibilities for viral infections subsequently exacerbating or initiating the symptoms of psoriasis. The research provided a comprehensive assessment of how viral infections and inflammatory mediators (i.e., resistin) can mediate the pathophysiology of psoriasis.

### 2.2. Inclusion and Exclusion Criteria

A group of 90 psoriasis patients from November 2022 to October 2023 who gave informed consent and provided a complete medical and demographic background were included in the study. Institutional Ethical committee approval was obtained for the project (ECR/300/Insst/AP/2013/RR-19). Patients were diagnosed with psoriasis based on clinical and/or histopathological evaluation and were in the age group of 18–65 years. The study included patients who were willing to provide informed consent and those who had not received systemic therapy for psoriasis or antiviral treatment in the last three months. Individuals with other autoimmune or systemic inflammatory disorders, as well as those currently undergoing immunosuppressive therapy or biologic treatments for psoriasis, were excluded from the study. Additionally, patients with a documented history of chronic systemic conditions were excluded from the study and were also not considered eligible for participation if they adhered to the following conditions: having infections unrelated to the study (e.g., tuberculosis), pregnant or lactating women, individuals unable or unwilling to provide informed consent, patients with severe comorbid conditions (e.g., advanced liver or kidney disease) that could confound the results, and individuals with recent vaccination (within the last three months) against viral infections being studied.

### 2.3. Sample Collection and Preparation

This study involved collecting venous blood samples from patients with psoriasis. The blood samples were centrifuged at 3000 rpm for 10 min to isolate the serum, which was subsequently aliquoted and stored at −20 °C for future analysis.

#### Detection of CRP Levels

To measure CRP (C-reactive protein) levels, serial dilutions of the test sample were prepared using isotonic saline at concentrations of 1:2, 1:4, 1:8, 1:16, 1:32, 1:64, etc., based on the qualitative method. A small amount from each dilution was placed onto separate reaction circles. One drop of the CRP latex reagent, provided with the kit, was added to each dilution, and the mixture was gently stirred using a stick. Agglutination was observed macroscopically by rocking the slide back and forth for two minutes, using a stopwatch. The CRP concentration was calculated by multiplying the reagent sensitivity (0.6 mg/dL) by the highest dilution of serum that showed visible agglutination.

### 2.4. ELISA (Enzyme-Linked Immuno Sorbent Assay)

Human Resistin (RETN GENLISA) was used for the quantitative detection of resistin levels, employing the sandwich ELISA method. In this technique, microwells were precoated with human Resistin monoclonal antibodies. The sample and standard solutions were then added, allowing the resistin present in the sample to bind with the antibodies. Following this, a biotin-labeled RETN antibody was introduced, followed by Streptavidin-HRP. The mixture was protected for 1 h at 37 °C to form a complex. Any non-specific binding was eliminated by washing with a wash buffer.

For HSV-1 and HSV-2, the ELISA test utilizes an IgM capture method. First, IgM antibodies present in the sample are captured by solid-coated anti-IgM antibodies on the wells. After washing away additional components of the sample, specific IgM antibodies are detected by adding a purified conjugate of HSV-1 and 2, labeled with a specific antibody conjugated to HRP. Unbound conjugates are washed away, and a chromogen is added. The optical density is directly proportional to the concentration of HSV-1 and HSV-2 IgM antibodies in the sample, which is measured at 450 nm/620–630 nm using an ELISA reader. The Microlisa HIV ELISA test, an indirect detection method, employs HIV-1 envelope proteins (gp41, the C-terminus of gp120) and HIV-2 gp36, which are immunodominant epitopes, coated onto microtiter wells. If antibodies against HIV-1 or HIV-2 are present in the sample, they will bind to the corresponding antigens immobilized on the wells of the ELISA plate. After washing to remove unbound substances, horseradish peroxidase (HRP)-conjugated anti-human IgG is added, which binds to the HIV antigen–antibody complex. The HCV Microlisa test utilizes a mixture of antigens representing both structural and non-structural components of the virus, including CORE, E1, E2, NS3, NS4, and NS5.

The principle behind the HEPALISA test is the “Direct Sandwich” method, where microwells are coated with monoclonal antibodies. Samples are added alongside an enzyme conjugate, forming a sandwich complex in which the HBsAg is trapped between the antibody and the HRP-conjugated antibody. Any unbound conjugate is washed away, and the amount of peroxidase bound is proportional to the concentration of HBsAg present in the sample. The color change is observed after the addition of the substrate.

For the HZ antibody assays, the manufacturer’s protocol was followed. Typically, 5 to 10 microliters of serum samples, standards, and controls were added to the wells of 96-well microplates pre-coated with the respective antigen or antibody. The plates were incubated at 37 °C for 30 to 90 min, as per the assay protocol. After incubation, the wells were washed 4 to 6 times with wash buffer to remove unbound materials. Enzyme-conjugated antibodies were then added, and the plates were incubated for an additional 30 to 60 min at 37° C. Next, a chromogenic substrate (TMB) was added and incubated in the dark for 10 to 15 min at either room temperature or 37 °C. The enzymatic reaction was stopped by adding a stop solution (1N H_2_SO_4_), and absorbance was measured at 420–440 nm with a reference wavelength of 620–630 nm using a microplate reader, following the manufacturers’ instructions for each specific ELISA kit.

### 2.5. Data Analysis

Absorbance readings were interpreted in accordance with the manufacturers’ guidelines for each respective kit. Cut-off values were calculated individually for each kit based on the manufacturers’ instructions to determine seropositivity.

## 3. Results

**Figure 2:**
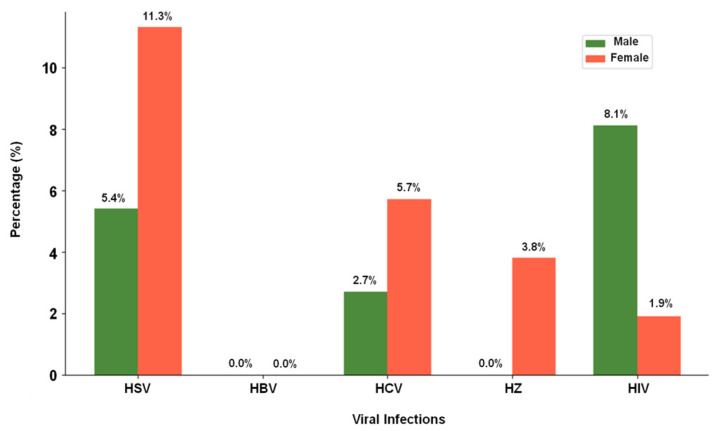
Presentation of gender-based distribution of viral infections.

In a study conducted on 90 psoriatic patients aged between 23 and 45 years, the sample population consisted of 53 females (58.9%) and 37 males (41.1%). Among these patients, 62 individuals (68.9%) exhibited a worsening of their psoriasis symptoms. An important finding of the study was the detection of HSV antibodies in eight samples (8.9%), suggesting that nearly one-tenth of the participants had an active or recent HSV infection. Notably, female patients had a higher prevalence of HSV, HCV, and HZ antibodies compared to males. In the male group, 5.4% tested positive for HSV, while in females, the percentage was higher at 11.3%. Similarly, the prevalence of HCV was observed to be 2.7% in males and 5.7% in females, while HZ antibodies were detected in 3.8% of females, but none of the male participants tested positive for HZ (Table 1 and Figure 2). This finding points to potential gender differences in susceptibility or exposure to these infections in individuals with psoriasis.

**Figure 3:**
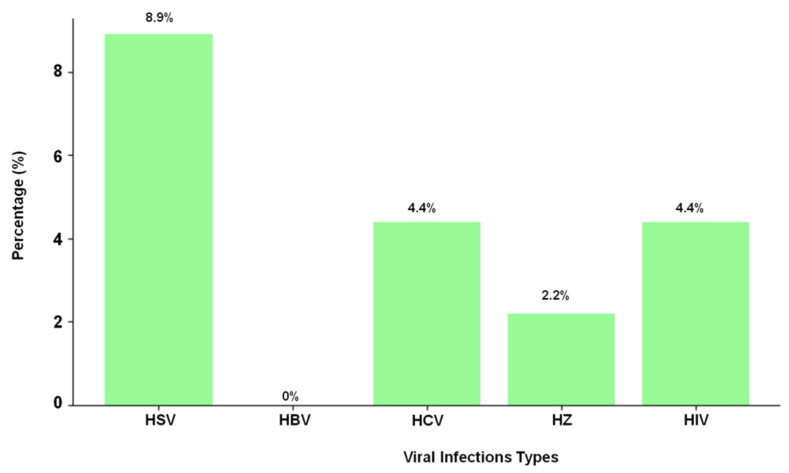
Presentation of viral infection in psoriasis patients.

In addition to HSV, four samples (4.4%) tested positive for HIV antibodies, and four samples (4.4%) tested positive for HCV antibodies (Table 2 and Figure 3). The presence of HIV antibodies is significant, as it is consistent with established research that links HIV infection to more severe psoriasis. The impact of HIV on immune regulation is known to exacerbate psoriasis symptoms, suggesting that the immune dysregulation caused by the virus contributes to increased psoriasis severity in affected individuals. Among the participants, HIV co-infection was more prevalent in males, with 8.1% of male patients testing positive for HIV antibodies, compared to only 1.9% in females.

Interestingly, the study found that none of the samples tested positive for HBsAg, indicating that there were no active hepatitis B infections present in the study population. This suggests that, while other viral infections were prevalent in psoriatic patients, hepatitis B was not a concern in this cohort.

A high prevalence of viral infections (HSV and HIV) was observed with high resistin levels (>35 ng/mL), compared to low resistin levels (Table 3).

The highest prevalence for HSV was seen in the elevated resistin group (14%) with a statistically significant trend (*p* = 0.002), suggesting that higher resistin could correlate with HSV infection. HIV has 8% prevalence in the elevated resistin group, with a statistically significant trend (*p* = 0.018), implying resistin may play a role in HIV susceptibility. No clear trends were reported for HCV, HZ, and HBV.

The findings of this study highlight the high prevalence of viral infections, particularly HSV, HCV, and HIV, among individuals with psoriasis. These infections appear to contribute significantly to the exacerbation of psoriasis symptoms, emphasizing the need for careful monitoring of viral co-infections in these patients. This study offers important insights into the intricate interplay between psoriasis and viral infections, underscoring the need for comprehensive healthcare management for psoriasis patients, especially those with co-existing viral infections.

## 4. Discussion

RIG-I (Retinoic acid-inducible gene I) is a key player in the detection of RNA viruses. When viruses evade RIG-I-mediated antiviral signaling, it can lead to persistent viral infections that induce the expression of IL-23 in CD11c+ dendritic cells (DCs) in individuals with a genetic predisposition. This immune response plays a significant role in the development of psoriasis, contributing to the inflammatory processes that characterize the condition [49]. HIV co-infection was detected in 4.4% of the study participants, which is consistent with the well-established link between HIV and psoriasis severity. Psoriasis is more severe in HIV-positive individuals, primarily due to the virus’s impact on immune regulation. Direct triggering of psoriasis by HIV is a result of superantigens or costimulatory factors provided in antigen presentation [43]. This causes elevated IFN-γ production by activated CD8+ T cells [50]. HIV-infected immune cells release the neuropeptide substance P, which modulates inflammatory and immune responses and stimulates keratinocyte proliferation [51]. The depletion of CD4+ T cells in HIV patients disrupts the immunological balance, perhaps favoring pro-inflammatory pathways mediated by Th17 cells, which are linked to psoriasis pathogenesis. In contrast, Morar et al. (2010) [43], depending on the cohort, showed a higher incidence of psoriasis in HIV-positive people ranging from 6% to 10% [44,52]. The differences in regional HIV prevalence or the strict inclusion criteria of our study, which may have excluded more advanced HIV cases, could be the factor for lower prevalence in our study.

The link between HCV infection and psoriasis shows that 4.4% of patients have detectable HCV antibodies. Chronic HCV infection is associated with systemic inflammation and immune dysregulation, which may worsen psoriasis. HCV induces the production of inflammatory cytokines such as cathelicidin, TLR9, IFNγ, TNF-α, and IL-6, all of which are key contributors to psoriasis pathogenesis [53]. Additionally, molecular mimicry between HCV proteins and self-antigens may play a role in promoting autoimmunity. A study by Noe et al. (2017) [40] found that the prevalence of HCV in psoriasis patients is higher, typically ranging from 5% to 10%, compared to the general population, with variation based on geographic and population-specific factors [54]. The greater prevalence of HSV, HCV, and HZ antibodies in female patients than in males may be attributed to hormonal differences or gender-related immune response variations. The present findings from this study show less prevalence than the previously reported studies, which could be the regional differences in HCV transmission and prevalence. Directly active antiviral agents had significantly better outcomes compared to other treatment modalities in eradicating HCV, with better psoriasis resolution [55]. Following HCV eradication with these agents, immune recovery was observed, characterized by a decrease in co-inhibitory molecules like TIGIT, an increase in HCV-specific CD8+ T cells, and a reduction in peripheral NK cells [56]. This led to a significant improvement in the pro-inflammatory state, benefiting both psoriasis and HCV outcomes [57,58] A study by Bu, J. et al. in a U.S. population survey showed no significant difference in the prevalence of hepatitis B or C between individuals with and without psoriasis [59].

The present study shows 2.2% positivity (2 in 90 psoriatic patients) for HZ infection, consistent with the previous reports from Tsai et al., 2017 and Ting, S.W. et al., 2021 [46,60]. Tsai et al. reported 1.41% positivity of HZ infection in psoriatic patients compared to the non-psoriatic population, whereas Singer, D. et al. reported 1.21% positivity [46,61]. It is also evident from Ting, S.W. et al. that the psoriatic patients receiving the biological therapy show higher HZ infection rates compared to the psoriatic patients receiving conventional therapy [60]. A retrospective observational study by Megna, M. et al. found no significant increase in the risk of developing herpes zoster among psoriasis patients undergoing treatment with biologics or traditional systemic therapies [62]. A sudden withdrawal of oral corticosteroid (prednisolone) causing disseminated papulovesicular eruptions similar to VZ has been reported by Garg et al. (2012) [63]. HSV I & II PCR was negative, with histopathology revealing a balloon. The patient responded to Acyclovir with a resolution of the eruptions while psoriasis treatment continued. A high risk of HZ is observed when the comparison is performed between the psoriasis and non-psoriasis female cohort groups. Volunteers studied were in the age group of 20–39 years with psoriasis and without comorbidities [32]. VZV is known for its affinity to T lymphocytes, which, on activation, release infectious virions from CD3+T lymphocytes apart from CD4+ and CD8+ cells [64]. Proteins like cutaneous leukocyte antigen (CLA) and the expression of C-C chemokine receptor 4 (CCR4) from infected CD4+ T lymphocytes acting as memory T-cells explain its cutaneous affinity [65]. In psoriasis, increased infiltration by CD4+ T lymphocytes is already elaborated in many studies apart from CD8+ T lymphocytes by CXCL16-CXCR6 associations [66,67,68,69]. A possible role of resistin, along with other inflammatory mediators, is proposed in this pathology, which can be reduced by treating the viral infections, thereby breaking the vicious cycle of inflammation. The present results also emphasize the importance of HZ management (immunization and symptomatic management) in patients receiving biologic therapy.

Significant clinical improvement of psoriasis was observed in an inactive chronic HBV patient with positive HBsAg, HBsAb of <5 mlU/mL, positive total HBcAb, and a low HB viral DNA level of 10 IU/mL with tenofovir, an antiviral drug used to treat HBV, reported by Chung et al. in 2023 [70]. Psoriasis improved with a BSA~1% and PGA 2, with apparent post-inflammatory hyperpigmentation on her extremities in areas of resolved plaques. In the present study, zero HBV positivity was observed among the psoriatic patients. This finding contradicts the previous reports by Suh et al. [71] and Kanada et al. [72] where the prevalence of HBV is <3%. The results could be regional differences or vaccination coverage in this area.

A study by Fedorova, U.V. et al. found that in psoriasis patients who are HSV positive, there is a 3.5-fold increase in IFN-α synthesis in serum compared to healthy individuals, and a 2.8-fold increase compared to psoriasis patients without HSV [73]. In psoriasis patients, 1 in 6 persons in the US have an HSV-2 seropositive status, with African American women having the highest frequency [74]. The research reported psoriasis reappearance in a female with a genital herpes flare-up confirmed by punch biopsy and culture for HSV 2, which showed resolution on being treated by Acyclovir within a week. HSV suppression reduced both the viral outbreaks and the perimenstrual exacerbation of psoriasis in the patient. Given the high prevalence of genital HSV infections, particularly subclinical ones, it is plausible that HSV plays a role in the worsening of psoriasis and contributes to the exacerbation of psoriasis, and treatment of it positively impacts the resolution of psoriasis [75].

Out of a total of 90 patients included in the research, 68.9% experienced worsening psoriatic features. This is an important finding as it can suggest a potential link between psoriatic inflammation and viral infections, which can promote features of psoriasis. Furthermore, the prevalence of viral infection rates in these patients was markedly high. HSV was detected in 8.9% of patients, while HCV and HIV were detected in 4.4% of patients each. Therefore, viral co-infection appears to be a factor influencing the course of both the psoriasis and the immune response. One of the key results from the analysis was the strong association of elevated resistin levels with HSV and HIV infections. This was supported by statistically significant findings showing higher levels of resistin in positive HSV seropositivity (*p* = 0.002) and HIV positivity (*p* = 0.018), suggesting that these viral infections can play a role in the modulation of resistin levels, a biomarker for proinflammation. Resistin, which appears to have a role in modulating immune responses and inflammation, can therefore be a useful biomarker for understanding how viral infections impact the clinical progression of psoriasis and other inflammatory diseases. Furthermore, the research found that patients with higher resistin levels demonstrated a trend of having an increased number of viral co-infections. This finding indicates that resistin can potentially not only be a by-product of a single viral infection but also indicative of broader systemic inflammation that can predispose patients to multiple viral infections. This provides a strong rationale for reviewing both viral serologies and inflammatory biomarkers like resistin in a patient with worsening psoriasis.

The research link between resistin and viral seropositivity in psoriasis is important, as it improves understanding of how viral infections exacerbate psoriasis. Viral infections such as HSV can aggravate psoriasis through the activation of immune responses. This inflammatory process can involve resistance. Similarly, HZ is linked to the reactivation of the varicella-zoster virus and can exacerbate psoriasis. The role of resistin in causing exacerbation of HZ is unknown. HBV and HCV can cause systemic inflammation, which can worsen psoriasis. Measuring resistin levels in individuals with HBV or HCV can help understand whether these viruses can worsen the severity of psoriasis. In individuals living with HIV, it has been suggested that the dysfunction of the immune system can add to the severity of psoriasis and resistance can impact immune responses that affect psoriasis. These viral infections in conjunction with the examination of resistin can help provide useful information for diagnostic, treatment, and management options for psoriasis, particularly in individuals who have viral infections existing alone with psoriasis.

- Practical Considerations in Patient Care

Determining resistin levels in psoriasis patients has a good amount of scientific value and practical utility. Scientifically, the level of resistance in the body can help clarify some of the biological inflammatory mechanisms involved in the pathogenesis of psoriasis. Because resistin is a pro-inflammatory cytokine and influences the regulation of immune responses, the concentration of resistin in the body can help users understand the degree of inflammation present. Understanding how resistin interacts with and affects viral infections can provide additional clarity into how viral triggers, such as HSV and HIV, influence psoriasis symptoms and disease trajectory. From a practical standpoint, measuring resistin levels can provide clinicians with valuable information to help determine whether patients are at increased risk for viral co-infection. The use of resistin as a biomarker to identify patients is a proactive strategy because the results can help clinicians monitor psoriasis patients for significant increases in viral co-infections, such as HSV or HIV, while offering a more specific treatment for patients at high risk of suffering from concomitant viral infections. This represents an excellent opportunity for personalized treatment options for clinicians who are utilizing therapeutic tools more effectively. Finally, resistin could identify high-risk patients before they developed significant complications as a result of this viral co-infection. This can be instrumental in the effective management of the disease and restoring better control over psoriasis, especially when the patient has a co-infection that promotes the exacerbation of symptoms.

## 5. Conclusions

Since both psoriasis and viral infection involve significant immune activation and chronic inflammation, there is a need to investigate the prevalence of viral infections in psoriasis patients with elevated resistin and CRP levels. High levels of these markers in psoriasis patients may suggest a heightened inflammatory state that could be worsened by concurrent viral infections. Without thorough screening, viral infections may be misdiagnosed or underdiagnosed in these patients, leading to suboptimal therapy for both diseases. The research supports the conclusion that viral infections (especially HSV and HIV) are closely associated with increased resistin levels in patients with psoriasis. From 90 patients, 68.9% reported aggravated psoriasis flares, and the prevalence of viral infection was significant for HSV (8.9%), HCV (4.4%), and HIV (4.4%). Increased resistin levels were significantly associated with both HSV (*p* = 0.002) and HIV (*p* = 0.018) positivity, in which it is proposed that these viral infections can influence the regulation of resistin (a pro-inflammatory cytokine) and worsen psoriasis exacerbation. The highest prevalence of viral co-infections was in patients with increased resistin levels, illustrating the association of resistin with inflammation and viral activity in psoriasis. The research reinforces the need to find viral co-infections as part of the management of psoriasis, as elevated resistin can potentially impact the severity of the disease, supporting the premise that controlling viral infections can be essential for controlling the disease progression of psoriasis. Viral infections should be monitored continuously, not only with the initial screening of the patient but also during treatment, as flare-ups may occur after prolonged periods of remission. Consequently, monitoring can be undertaken periodically, because even if the infected patient is free of symptoms, viral infections can be dormant and possibly reactivate, potentially leading to reactive psoriasis flare-ups and unfavorable responses to treatment. Continuous monitoring can also allow a timely call to action or allow for necessary modifications to treatment plans when either psoriasis or underlying viral infections require attention. The presence of viral infection in psoriasis patients could lead to the progression and increased severity of psoriasis. Detecting the prevalence of viral infections among psoriatic patients is helpful in efficient patient management and treatment methods. The findings also help in recommending screening strategies and improving public health efforts. A study to evaluate the resistance in psoriatic lesions will help understand the complex pathogenesis of viral infection flare-ups. Ultimately, identifying the viral infections in skin diseases emphasizes the importance of integrated patient care management.

## Figures and Tables

**Table 1:** Gender-based distribution of viral infections.

Viral Infection	Male (*n* = 37)	Female (*n* = 53)
HSV	2 (5.4%)	6 (11.3%)
HBV	0 (0%)	0 (0%)
HCV	1 (2.7%)	3 (5.7%)
HZ	0 (0%)	2 (3.8%)
HIV	3 (8.1%)	1 (1.9%)

**Table 2:** Prevalence of viral infection in psoriasis patients.

Viral Infection	Number of Cases (*n* = 90)	Percentage %
HSV	8	8.9%
HBV	0	0%
HCV	4	4.4%
HZ	2	2.2%
HIV	4	4.4%

**Table 3:** Viral co-infection prevalence by resistin levels in psoriasis patients (*n* = 90).

Viral Infection	Elevated Resistin (12–20 ng/mL) (*n* = 50)	Normal Resistin (4–12 ng/mL) (*n* = 30)	Low Resistin (<3–4 ng/mL) (*n* = 10)	*p*-Value (Trend Test)
HSV	7 (14.0%)	1 (3.3%)	0 (0%)	0.002
HIV	4 (8.0%)	0 (0%)	0 (0%)	0.018
HCV	1 (2.0%)	2 (6.7%)	1 (10.0%)	0.25
HZ	0 (0%)	1 (3.3%)	1 (10.0%)	0.12
HBV	0 (0%)	0 (0%)	0 (0%)	N/A

N/A: Not applicable.

## Data Availability

The data presented in this study are available on request from the corresponding author due to ethical restrictions.
